# Kufor–Rakeb Syndrome in a Guatemalan Patient With an *ATP13A2* Gene Pathogenic Variant: A Case Report

**DOI:** 10.1155/crig/6993134

**Published:** 2025-11-18

**Authors:** Rebeca Méndez-Veras, Allan Urbizo, Julio Cabrera, Suzette Boburg

**Affiliations:** ^1^Instituto de Investigaciones Químicas, Biológicas, Biomédicas y Biofísicas (I2QB3), Universidad Mariano Gálvez, Guatemala City, Guatemala; ^2^Facultad de Medicina, Universidad Francisco Marroquín, Guatemala City, Guatemala

**Keywords:** *ATP13A2*, autophagy–lysosome pathway, juvenile-onset parkinsonism, Kufor–Rakeb syndrome

## Abstract

Parkinson's disease (PD) is a neurodegenerative condition characterized by progressive loss of dopaminergic neurons and by heterogeneous etiologies and clinical manifestations. Juvenile‐onset forms are rare and can be caused by biallelic mutations in several genes. Kufor–Rakeb syndrome (KRS) is an autosomal-recessive form of early-onset parkinsonism caused by pathogenic variants in the *ATP13A2* (*PARK9*) gene. This P5B-ATPase dysfunction impairs lysosomal processing, leading to the accumulation of *α*-synuclein. Here, we present the first documented Guatemalan case of KRS, a young woman with progressive motor and cognitive decline. Genetic testing identified a homozygous pathogenic variant in *ATP13A2*. This report underscores the importance of recognizing KRS in diverse populations and of using gene-based testing to guide diagnosis, counseling, and multidisciplinary supportive care.

## 1. Introduction

Parkinson's disease (PD) affects approximately two individuals per one thousand in the general population, with its prevalence increasing to about 3% among people over 80 years of age [1]. The classic motor phenotype involves bradykinesia, rigidity, and resting tremor, although patients commonly experience an array of nonmotor manifestations, including dysautonomia, cognitive impairment, and psychosis [2].

PD and related parkinsonian syndromes may be sporadic or monogenic. Autosomal-dominant causes include mutations in *LRRK2* and *SNCA*, whereas autosomal-recessive forms are linked to genes such as *PARK2* (*Parkin*), *PINK1*, *DJ-1*, *PLA2G6*, *FBXO7*, *DNAJC6*, *SYNJ1*, *VPS13C*, and *ATP13A2* [3, 4]. Among these, *ATP13A2* encodes a lysosome-related P-type ATPase; dysfunction of this transmembrane protein impairs lysosomal autophagy and promotes intracellular accumulation of *α*-synuclein [5, 6]. Pathogenic variants in *ATP13A2* define Kufor–Rakeb syndrome (KRS) and are distinct from other *PARK* genes, which engage in ubiquitin-proteasome pathways, mitochondrial quality control, or synaptic vesicle recycling [3–5].

The *ATP13A2* locus lies on Chromosome 1p36 and encodes a P5B-ATPase that transports polyamines across lysosomal membranes. Loss-of-function variants cause misfolding of the protein, retention in the endoplasmic reticulum, and subsequent degradation via the endoplasmic reticulum–associated degradation (ERAD) pathway [5]. Deficient *ATP13A2* activity reduces lysosomal polyamine export and compromises autophagy, leading to toxic accumulation of *α*-synuclein and eventual dopaminergic neuron loss [6–8]. Clinically, KRS presents before age 20 with a combination of parkinsonian symptoms, spastic paraparesis, supranuclear gaze palsy, cerebellar signs, and early cognitive decline. Given its genetic rarity, fewer than 50 individuals worldwide have been reported with confirmed KRS [9–11].

We present the case of a Guatemalan woman with progressive neuropsychiatric and motor decline in whom a targeted neuromuscular gene panel identified a homozygous pathogenic variant in *ATP13A2*. To our knowledge, this is the first description of a pathogenic *ATP13A2* variant in a patient from Guatemala and adds to the growing geographic diversity of KRS.

## 2. Case Presentation

The patient was a 21-year-old woman born at term via caesarean section to nonconsanguineous parents in Retalhuleu, Guatemala. Perinatal history was unremarkable, and she achieved age-appropriate developmental milestones without delays. There was no family history of parkinsonism, movement disorders, or other neurodegenerative diseases.

At 9 years of age, she experienced hepatitis following dengue fever. By age 10, she developed bradyphasia (slowed speech) and dysphagia (difficulty swallowing) as well as cognitive disengagement characterized by decreased environmental awareness, prolonged introspective episodes, and attention deficits. Over the following year, she exhibited progressive dysphagia and masticatory difficulties that led to severe weight loss and cervical muscle weakness, compromising head support; ptosis was also observed. By early adulthood (20 years), she had lost all of her teeth, which severely limited her nutritional intake. Although edentulism is not a recognized feature of KRS, we attribute it in this case to profound malnutrition and inadequate dental care.

Speech impairment was one of the earliest signs of her neurodegeneration. Family members first noted difficulty articulating words at age 10. This impairment progressed steadily throughout adolescence, leading to largely unintelligible speech by her late teens. At the time of evaluation (age 21), the patient was entirely nonverbal (mutism) and relied exclusively on gestures for communication. Assessment of her language revealed that her receptive language was limited, as evidenced by her inability to consistently follow simple instructions. Furthermore, she displayed significant cognitive impairment, reflecting a loss of previously acquired cognitive functions rather than a delay in developmental milestones.

The patient's mobility was severely restricted, necessitating the use of a wheelchair for short-distance ambulation. She displayed marked axial rigidity and postural instability, coupled with profound muscle wasting secondary to severe malnutrition. Photophobia and oculomotor dysfunction were also reported. Her persistent dysphagia required prolonged periods in a recumbent position.

Behavioral disturbances constituted a significant component of her clinical presentation. These included episodes of irritability, unpredictable rage outbursts, and a generally labile mood. These neuropsychiatric symptoms often appeared to cluster around hormonal fluctuations, such as menarche or dysphoric mood states, which could last for an hour or two. Indicated by a neurologist, the patient was treated empirically with carbamazepine and corticosteroids for a 1-month period, though this regimen yielded no improvement in her condition. Because her presentation suggested a hereditary neuromuscular disorder, a targeted next-generation sequencing panel for neuromuscular diseases was requested but could not be performed at the moment because of economic restraints.

**Figure 1:**
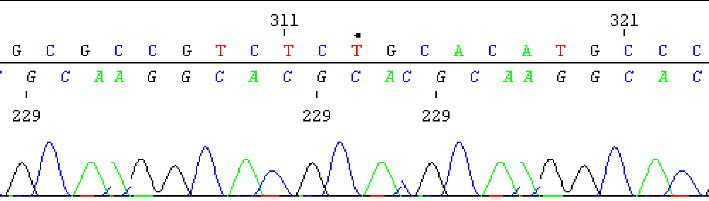
Sanger sequencing of the proband showing a homozygous ATP13A2 c.2629G>A missense variant.

When the family had economic possibilities, they approached our laboratory to request the NGS panel for neuromuscular diseases. This was 11 years later than indicated by the geneticist. Given the presentation suggestive of a hereditary neuromuscular disorder, a targeted next-generation sequencing panel for neuromuscular diseases (TruSight One, Illumina) was employed. This sequencing identified a homozygous missense variant in the *ATP13A2* gene: c.2629G>A, p.(Gly877Arg), NM_022089.4. This finding was subsequently confirmed using Sanger sequencing (Figure 1). Parental testing to confirm carrier status could not be performed due to financial constraints.

Sanger sequencing shows c.2629G>A substitution in ATP13A2.

The relationship between the patient's symptoms and the genetic findings is summarized in Table 1.

## 3. Discussion

This report describes a 21-year-old woman with early-onset parkinsonism, spasticity, cognitive decline, and behavioral disturbances. The proband's phenotype and diagnostic timeline are summarized in Table 1. Genetic testing revealed a homozygous pathogenic variant in *ATP13A2* (c.2629G>A; p.(Gly877Arg)) predicted to result in loss of function and consistent with KRS (*PARK9*). Fewer than 50 individuals with KRS have been reported worldwide, and most pathogenic variants are loss-of-function alterations [4]. Our case adds to the phenotypic and geographic spectrum of KRS and underscores the diagnostic value of gene panels in young patients presenting with unexplained symptoms related to parkinsonism.

The sequencing assay used in our patient targeted genes associated with heritable neuromuscular disorders rather than representing a comprehensive neurologic panel. Nevertheless, it successfully identified the causative mutation. After confirmation of the genetic diagnosis, we counseled the family on the natural history of KRS and the lack of disease-modifying therapies. Supportive management focuses on nutritional optimization, physiotherapy, and monitoring for complications. The profound tooth loss observed in our patient is unusual in KRS and likely reflects severe malnutrition and limited dental access; clinicians should consider comorbid conditions when interpreting atypical features.

Psychiatric manifestations are increasingly recognized in KRS. Reports have described episodes of aggression, hallucinations, and psychosis, but experience with antipsychotic therapy remains limited [12]. Given our patient's episodic agitation and inability to articulate internal experiences, psychosis cannot be excluded and may warrant cautious pharmacologic intervention.

This case highlights several important considerations. First, the onset of speech impairment in KRS may occur years before frank mutism, underscoring the need for early speech therapy and augmentative communication strategies (AAC). Second, cognitive impairment in KRS reflects neurodegeneration rather than developmental delay; clinicians should use accurate terminology when counseling families. Third, behavioral disturbances should prompt evaluation for comorbid psychiatric illness and treatable triggers. Finally, documenting cases from diverse populations broadens our understanding of the disorder's genetic heterogeneity and informs regional genetic counseling.

This report describes the first documented instance of a pathogenic *ATP13A2* (*PARK9*) variant in a patient from Guatemala, adding crucial data regarding the geographic and phenotypic scope of KRS. The patient exhibited the classic severe juvenile-onset parkinsonism, pyramidal signs, and early cognitive impairment, complicated by profound secondary issues such as malnutrition and edentulism.

Although our patient's edentulism occurred early, it likely resulted from chronic malnutrition and inadequate oral care rather than being a primary manifestation of KRS or juvenile-onset parkinsonism. Epidemiological evidence linking oral health and PD remains mixed. A large Korean cohort study reported that severe tooth loss (≥ 15 teeth) was associated with a 38% increase in the risk of new-onset PD, whereas frequent tooth brushing and regular dental visits were associated with lower risk [13]. A Taiwanese nested case-control study found that individuals aged 40–69 without periodontal inflammatory disease who underwent dental scaling for five consecutive years had a substantially reduced risk of PD, and a matched cohort study showed that periodontal inflammatory disease increased PD risk [14, 15]. Another systematic review and meta-analysis concluded that there is no bidirectional association between periodontitis and PD, although PD patients often have worse periodontal status [16]. These studies suggest that tooth loss and periodontal disease may contribute to systemic inflammation that potentially exacerbates neurodegeneration but are not causative features of juvenile parkinsonism or *ATP13A2*-related disease. Early dental interventions, nutritional support, and preservation of natural dentition are crucial to minimize systemic inflammation and maintain cognitive function [17].

The successful diagnosis using a targeted neuromuscular gene panel highlights the necessity of considering *ATP13A2* variants in the differential diagnosis of complex hereditary neuromuscular syndromes. Given the patient's severe, progressive neuropsychiatric disturbances and inability to self-report, potential KRS-associated psychosis must be actively evaluated and managed.

The management of KRS requires an aggressive, multidisciplinary approach focused not only on the primary motor and cognitive deficits but also on preventing secondary complications and providing early, supportive care including AAC and targeted nutritional support.

This case report expands the phenotypic and geographic spectrum of KRS and underscores the need to consider *ATP13A2* variants in patients with severe juvenile-onset parkinsonism across Latin America. Documenting cases from diverse populations improves our understanding of disease variability, guides targeted genetic testing, and supports the timely initiation of multidisciplinary care. Enhanced awareness of KRS in Latin American countries may also facilitate surveillance studies, inform regional epidemiology, and encourage the inclusion of these populations in future therapeutic trials. By sharing this case, we hope to support global efforts to refine KRS diagnosis and management and to highlight the value of multidisciplinary care in neurodegenerative diseases.

## Figures and Tables

**Table 1:** Clinical phenotype and diagnostic timeline.

Domain	Symptom/finding	Age of onset/observation	Relevance to KRS (*PARK9*)
Speech/bulbar	Bradyphasia, dysphagia, mutism/nonverbal	Age 10 (onset)	Severe, progressive bulbar failure, characteristic of the disease
Cognitive/language	Significant cognitive impairment, limited receptive language	Age 10 (onset)	Reflects neurodegeneration
Motor	Axial rigidity, postural instability, severe muscle wasting	Progressive	Classic parkinsonian symptoms combined with pyramidal signs
Neuropsychiatric	Episodic rage outbursts, labile mood	Adolescence	Warrants urgent evaluation for KRS-associated psychosis
Atypical secondary	Severe malnutrition, edentulism	Early adulthood	Severe complication arising from bulbar failure and socioeconomic context
Genetic diagnosis	Homozygous *ATP13A2* (c.2629G>A; p.(Gly877Arg))	Age 21	Confirms juvenile-onset autosomal-recessive parkinsonism

*Note:* Clinical phenotype and diagnostic timeline of the proband with *ATP13A2*-related Kufor-Rakeb syndrome (KRS). Ages indicate first recognition or documentation of each domain; “Progressive” denotes ongoing deterioration rather than a discrete onset. Edentulism is interpreted as a secondary complication of severe malnutrition and bulbar dysfunction, not a primary feature of KRS; *PARK9* and *ATP13A2*.

Abbreviation: KRS = Kufor–Rakeb syndrome.

## Data Availability

The data supporting the findings of this study are available from the corresponding author upon reasonable request.
